# Effect of Powdered Shells of the Snail *Megalobulimus lopesi* on Secondary-Intention Wound Healing in an Animal Model

**DOI:** 10.1155/2015/120785

**Published:** 2015-03-02

**Authors:** Paulo Henrique Muleta Andrade, Eric Schmidt Rondon, Carlos Alexandre Carollo, Maria Lígia Rodrigues Macedo, Luiz Henrique Viana, Albert Schiaveto de Souza, Carolina Turatti Oliveira, Maria de Fatima Cepa Matos

**Affiliations:** ^1^Centro de Ciências Biológicas e da Saúde, Universidade Federal de Mato Grosso do Sul, 79070-900 Campo Grande, MS, Brazil; ^2^Faculdade de Medicina Veterinária e Zootecnia, Departamento de Medicina Veterinária, Universidade Federal de Mato Grosso do Sul, 79070-900 Campo Grande, MS, Brazil; ^3^Instituto de Química, Universidade Federal de Mato Grosso do Sul, 79074-460 Campo Grande, MS, Brazil

## Abstract

Topical administration of powdered shells of the land snail *Megalobulimus lopesi* was evaluated in Wistar rats for their healing activity in an excision wound model. The animals were distributed into three groups—G1 (control): no therapeutic intervention; G2 (vehicle controls): Lanette cream once daily; G3 (experimental animals): treated with powdered shells. Variables investigated were: wound area contraction, angiogenic activity, morphometric data, leukocytic inflammatory infiltrate, and total leukocyte count in peripheral blood. Thermogravimetric analysis and quantification and characterization of powdered shell proteins were also performed. Wound area on days 3, 7, and 14 was smaller in G3, besides presenting wound closure on day 21 for all these animals. Topical administration of the powdered shells also led to an increased number of vessels at the wound site, higher leukocyte counts in peripheral blood, and increased leukocytic inflammatory infiltrate. The results lend support to the southern Brazilian folk use of *M. lopesi* powdered shells, as shown by the enhanced secondary-intention healing achieved with their topical administration to wounds in rats. Topical administration caused inflammatory response modulation, crucial to accelerating the healing process, the chronification of which increases the risks of wound contamination by opportunistic pathogens.

## 1. Introduction

Nature is a primary source of effective medicinal agents, and folk medicine has been the basis for the development of a large number of drugs. Plasters, ointments, and wound dressings based on animal fat, for instance, have been in use for millennia [1–3].

Along the history, humans have searched in nature how to obtain resources for their basic needs [4]. Many years of observation and experimentation have provided medical knowledge in the use of natural products [5]. Around 60% of commercially available drugs are based on bioactive compounds extracted from natural sources [6]. A great number of these natural products have come to the market from the scientific study of remedies traditionally used by various cultures around the world [6]. Zootherapy is defined as healing human diseases using animals or animal-derived products [2]. Nowadays, the use of animals with medicinal properties is a common practice worldwide. In China, more than 1500 animals are used as medicine; in India 15 to 20% of the Ayurvedic medicine is based on animal-derived substances. In Brazil, 326 animal species are recorded with medicinal purposes [7], whereas 584 medicinal animal species are reported in Latin America [8, 9].

Wound healing is a complex process that involves inflammation, reepithelialization, angiogenesis, and formation of granulation tissue and interstitial matrix, as well as processes performed by specific cells, such as keratinocytes, fibroblasts, and endothelial cells [10, 11].

Tissue repair comprises essentially three phases: inflammation, during which homeostasis is restored; proliferation, where wound granulation, contraction, and reepithelialization take place; and remodeling (or resolution), which determines the strength and appearance of the scar tissue [10, 12, 13].

Current methods of wound treatment include debridement, antibiotic therapy, tissue graft, proteolytic enzymes, and angiogenic agents; yet none precludes unwanted outcomes, such as hypertrophic scars or keloids. Developing drugs based on compounds from natural sources can be desirable in terms of public health management, by providing low-cost treatment options for cutaneous wounds or burns [14, 15].

Inflammatory response is crucial for survival, providing organisms with the ability to eliminate damaged or necrotic tissue and fight invading microorganisms (Chignon-Sicard et al., 2012 [12, 16]).

Medicinal plants and animals have been used virtually in all cultures as a source of medicine [17]. The use of biological resources for medicinal purposes, however, is not restricted to human diseases treatment, being also widely used for the treatment of livestock diseases [18].

Like plants, animals have been a source of medicinal treatments since antiquity. Their presence in the pharmacopoeia of traditional populations is considered universal by many researchers. The hypothesis of universal zootherapy, for example, postulates that every human culture that has developed a medical system utilizes animals as a source of medicine. The ubiquity of animals in folk medicine is illustrated by studies of ethnobiology worldwide [19, 20]. These studies, which have attracted increasing academic interest in recent years, have found a great deal of diversity in the animals used for therapeutic purposes, including insects [21, 22], vertebrates [9, 23], and marine invertebrates [8, 24]. Studies of medicinal animals can assist in pharmacological screening and may serve both as a source of medicine and as a measure of economic value for these species [24].

Obtaining inflammatory response modulators from plant or animal sources is important for the development of new therapeutic agents, in addition to encouraging the conservation of biodiversity for future generations [11].

Marine shells are widely used for medicinal purposes in India, China, and other Eastern countries. The antipyretic, antimicrobial, and wound-healing properties of* Cypraea moneta* powdered shells, for instance, have been experimentally demonstrated in Wistar rats [25].

In Northeast Brazil, macerated shells of* Megalobulimus oblongus* are an ingredient in folk remedies for asthma, and plasters containing* Iphigenia brasiliensis* shells are employed to treat gum irritation in children. In the southern region of the country, powdered shells of* Megalobulimus lopesi* are employed in the treatment of skin burns and hemorrhoids [26–29].

*M. lopesi*, locally known as “caracol-gigante-da-boroceia,” is a pulmonate land snail found mostly in the Cerrado and Atlantic Forest biomes. A nocturnal herbivore, the species lives in damp environments, where it is often found buried in leaf litter or topsoil. The shell is fusiform, elongated, and depressed, with about 5 whorls. The species is threatened with extinction by competition from invasive giant African land snails (*Achatina fulica*) [30, 31].

Grounded in established knowledge of the mechanism of wound repair, while also drawing on evidence from folk medicine, this study evaluated the wound healing and tissue regenerative properties of an ointment containing powdered shells of* M. lopesi* applied to cutaneous wounds in rats. The angiogenic properties of the ointment and its influence on inflammatory cellular response were also investigated.

## 2. Materials and Methods

### 2.1. Shell Collection and Ointment Preparation

Live snails were purchased from a private breeder and euthanized in our laboratory, where the shells were detached from the bodies, washed with distilled water, and dipped into liquid nitrogen to inactivate enzymes and preserve the integrity of protein and organic structures. Once lyophilized, the shells were ground to a powder in an MA701/21 ball mill (Marconi). An ointment containing 50% of powdered shells was prepared using Lanette cream (Dermavita Indústria Cosmética) as a water-soluble anionic vehicle.

### 2.2. *In Vivo* Experimentation

The study employed seventy-two 45-day-old male Wistar rats (*Rattus norvegicus albinus*) with mean body weight of 145 ± 10 g reared at the Animal Facilities of the Universidade Federal de Mato Grosso do Sul (UFMS). The animals were individually housed in plastic cages and kept in an alternating 12-hour light-dark cycle in a temperature-controlled room at 23°C. Food and water were available* ad libitum*. The experiments and procedures were approved by the UFMS Research Ethics Committee (permit 440/2012).

For creation of a dorsal skin wound, the animals underwent a surgical procedure in compliance with an antisepsis protocol. The rats were initially anesthetized with an intramuscular injection of ketamine hydrochloride (25 mg kg^−1^) and xylazine hydrochloride (5 mg kg^−1^), followed by shaving of the dorsal region. A skin circle measuring 0.6 cm in diameter was then excised from the center of the shaved area using a metal punch, exposing the fascia of the paravertebral muscles. Hemostasis was performed by digital compression with sterile gauze.

Immediately after hemostasis, the animals were randomized into three groups (*n* = 24 each) as follows: controls (G1): no therapeutic intervention; vehicle-control group (G2): Lanette cream applied topically immediately after wounding and once daily throughout the experimental period; experimental group (G3): powdered shells mixed with Lanette cream, applied topically immediately after wounding and once daily throughout the experimental period.

At 3, 7, 14, and 21 days after wounding, six animals from each group were anesthetized as described above and euthanized for prompt collection of material for macroscopic examination, peripheral blood leukocyte count, and histological analysis, after which they were placed in a CO_2_ chamber purged with CO_2_ gas for 60 s, where they remained until cessation of vital signs (3 to 5 min). The wounds were monitored daily throughout the experimental period.

### 2.3. Pathoanatomical Examination

The wounds were macroscopically monitored for tissue contraction, which was evaluated by comparing the postoperative baseline areas against those measured at 3, 7, 14, and 21 days. Transverse and longitudinal diameters in the live animals were measured using digital 11237 calipers (Mitutoyo). The mean diameter values thus obtained were subjected to statistical analysis.

### 2.4. Histological Examination

A tissue section containing the surgical wound was excised from each animal using a number 15 scalpel blade, submerged in 10% formalin solution, and processed for light microscopy. After embedding in paraffin, 5 *μ*m thick cross sections were cut at the wound sites and stained with hematoxylin-eosin (HE). The slides were photographed using a Jenaval microscope (Zeiss) with an attached DFC425 camera (Leica) and interpreted by an examiner blind to the treatment protocol adopted for the slide being evaluated.

The parameters evaluated were as follows.

**Figure 1:**
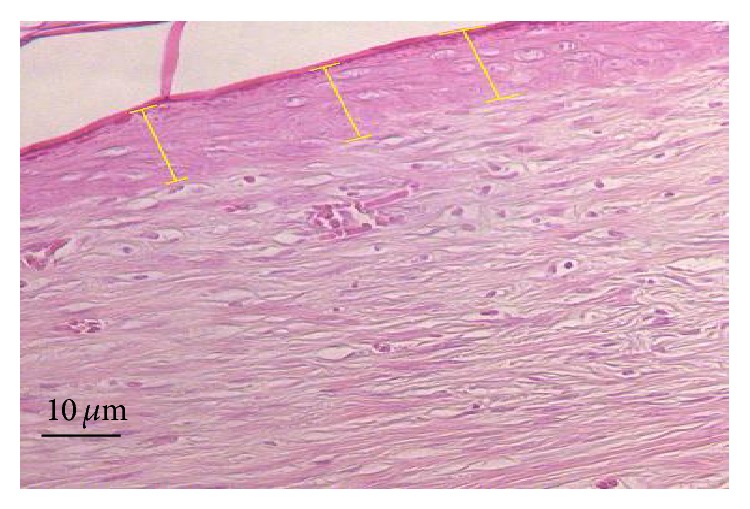
Morphometry of reepithelialized tissue formed at the excision site. Three measurements (*μ*m, yellow lines) were made in each optical field. Original magnification: 20x.

- Angiogenic activity: blood vessels present is 10 helds per slide, were counted at 7, 14, and 21 days using light microscopy at 20x magnification. Data were expressed as means for each wound.
- Morphometry: measurement of reepithelialized tissue thickness, using photomicrographs were captured with a videomicroscopy system and ImageJ 1.45 s software. Thickness was measured from the dermal junction to the outermost portion of the granular layer (Figure 1). For each animal, three randomized fields were examined at 20x magnification, with three measurements in each field. Data were expressed as means for each wound.
- Leukocytic inflammatory infiltrate: number of leukocytes per square micrometer of inflammatory infiltrate [32]. For each animal, 10 randomized fields (3305 *μ*m^2^ each) of HE-stained tissue were examined at 40x magnification, with three measurements in each field. Only fully visible cells were counted; those touching or partially cut off by the boundary lines were excluded. Data were expressed as means for each wound.

### 2.5. Leukocyte Count

For total leukocyte count, 20 *μ*L of blood was drawn from the orbital artery and added to 380 *μ*L of Turk's fluid (1 : 20) [33]. The results were expressed as cells per cubic millimeter.

### 2.6. Mineral Composition of Powdered Shells

Thermogravimetric analysis was performed on a Q50 thermogravimetric analyzer (TA Instruments) to quantify the principal minerals in the shells, using a heating protocol of 10°C min^−1^ up to 900°C in a nitrogen atmosphere at 70 mL min^−1^. To this end, the material was dissolved in chloroform and the resulting supernatant and sediment were analyzed separately.

### 2.7. Protein Quantification

Protein content was quantified as proposed by Bradford [34], using bovine serum albumin (BSA) as the standard protein.

### 2.8. 12.5% SDS-PAGE Electrophoresis

Sodium dodecylsulfate-polyacrylamide gel electrophoresis (SDS-PAGE) was performed as described by Laemmli [35]. The polyacrylamide plates were coated with a 5% stacking gel over a 12.5% running gel. The running gel was prepared with 1.5 M Tris-HCl buffer at pH 8.8, containing 0.1% (v/v) of 20% SDS, 0.025% PSA, and 0.1% TEMED, with a final volume of 7 mL. After polymerization of the running gel, the stacking gel was prepared with 0.5 M Tris-HCl buffer at pH 6.8 containing 0.1% (v/v) of 20% SDS, 0.025% PSA, and 0.1% TEMED, with a final volume of 2 mL. Electrophoresis was performed on a Mighty Small II Mini Vertical system (Hoefer Scientific Instruments) with dual miniplates.

The sample was dissolved in sample buffer (0.08 M Tris-HCl at pH 6.8, 10% glycerol, 4% SDS, and 0.001% bromophenol blue). BSA (66 kDa), chicken ovalbumin (45 kDa), pepsin from porcine gastric mucosa (34.7 kDa), bovine trypsinogen (24 kDa), and bovine *β*-lactoglobulin (18.4 kDa) were the proteins used as molecular weight markers.

Electrophoresis was run under a constant 30 mA current at 150 V. The gel was stained with 0.25% Coomassie brilliant blue R-250, 40% methanol, and 10% acetic acid in distilled water for 2 h. Excess dye was removed by washing the plates in a destaining solution (30% methanol and 10% acetic acid in distilled water).

### 2.9. Statistical Analysis

Values were expressed as means ± standard deviations of the means (means ± SEM). Data analysis was performed by comparing the means of each group at the different time points and comparing the means between groups (two-way ANOVA). Tukey's post hoc test was applied to data on wound area contraction, total leukocyte counts, angiogenic activity, leukocytic inflammatory infiltration, and morphometric measurements. Values of *P* lower than 0.05 were considered statistically significant. SigmaStat software, version 3.5, was employed for the statistical analysis.

## 3. Results

The wounds remained clean, with no perceptible changes. No signs of self-injury or self-biting were observed at the wound sites. Management was identical for all groups, so as to expose all animals to the same stress level.

### 3.1. Wound Area

Group effect, time-point effect, and interaction between these factors (two-way ANOVA for repeated measures, *P* < 0.001) were observed across groups for the ratio between wound area and baseline wound area. Comparisons of time points within each group revealed significant decreases in this ratio (7, 14, and 21 days; Tukey's post hoc test; *P* < 0.05).

Comparisons across groups at 3 days revealed the area ratio to be smaller in G3 than in G1 and G2 (Tukey's post hoc test, *P* < 0.05) and smaller in G1 than in G2 (*P* < 0.05). At 7 days, no significant differences in this ratio were observed between G3 and G1 (*P* > 0.05); yet values for both of these groups were lower than those of G2 (*P* < 0.05). At 14 days, the area ratio was smaller in G3 than in G1 and G2 (*P* < 0.05), with no significant difference between the latter two groups (*P* > 0.05). At 21 days, wound closure was observed in all animals in G3, while those in G1 and G2 showed signs of injury, despite the absence of a significant difference between the latter two groups (*P* > 0.05) (Table 1).

### 3.2. Angiogenic Activity

Comparisons across groups showed that the increase in new vessels at 7 and 14 days was greater in G3 than in G1 and G2 (*P* < 0.05). At these time points, the increase in G2 animals was greater than that observed for G1 (*P* < 0.05). In G2 animals, the increase in new vessels at 14 and 21 days was greater than at 7 days (*P* < 0.05), with no significant difference between 14 and 21 days (*P* > 0.05).

Group effect, time-point effect, and interaction between these factors (two-way ANOVA for repeated measures, *P* < 0.001) were observed across groups for increase in number of new vessels per optical field at the wound site, relative to 3 days postoperatively. Comparisons of time points within each group revealed significant increases in the number of new vessels in the period spanning from 7 to 21 days postoperatively (Tukey's post hoc test, *P* < 0.05). In G1 and G3 animals, the increase in new vessels was greater at 14 days than at 7 days. At 21 days, the increase was greater than at 7 and 14 days, for both groups (*P* < 0.05). In G2 animals, the increase in new vessels at 14 and 21 days was greater than at 7 days (*P* < 0.05), with no significant difference between 14 and 21 days (*P* > 0.05).

**Figure 2:**
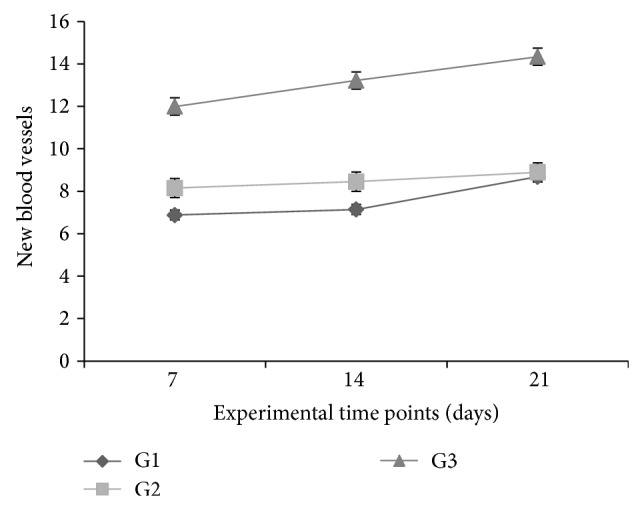
Increase in number of new vessels per optical field at wound site, counted at 7, 14, and 21 days, relative to 3 days postoperatively, by group. Values expressed as means ± SEM of groups (*n* = 6).

Comparisons across groups showed that the increase in number of new vessels at 7 and 14 days was greater in G3 than in G1 and G2 (*P* < 0.05). At these time points, the vessels increase was greater in G2 animals than for G1 (*P* < 0.05). At 21 days, the increase in new vessels was greater in G3 animals than in G1 and G2 (*P* < 0.05), with no significant difference between the latter two groups (*P* > 0.05) (Figure 2).

### 3.3. Morphometric Analysis of Reepithelialized Tissue

**Figure 3:**
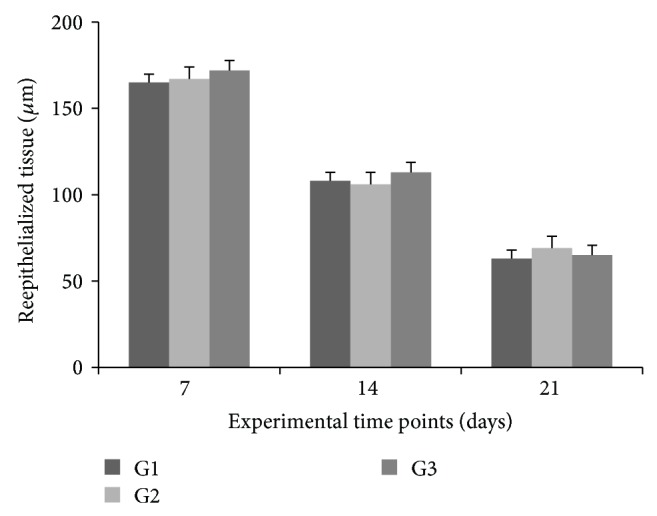
Reepithelialized tissue thickness at 7, 14, and 21 days, by group. Values expressed as means ± SEM of groups (*n* = 6).

A time-point effect (two-way ANOVA for repeated measures, *P* < 0.001) was observed for reepithelialized tissue thickness, but no significant differences were detected among groups (*P* > 0.05). Comparisons of time points within each group revealed significant decreases in reepithelialized tissue thickness from 7 to 14 to 21 days, in all groups (Tukey's post hoc test, *P* < 0.05) (Figure 3).

### 3.4. Leukocytic Inflammatory Infiltration

Group effect, time-point effect, and interaction between these factors (two-way ANOVA for repeated measures, *P* < 0.001) were observed across groups for leukocytic inflammatory infiltration, relative to baseline values.

Comparisons of time points within each group revealed gradual, significant increases in leukocytic inflammatory infiltration at 7 and 14 days, relative to 3 or 21 days (Tukey's post hoc test, *P* < 0.05). In G1 and G2 animals, leukocytic inflammatory infiltration increased significantly at 7 and 14 days, relative to 3 days (*P* < 0.05). At 21 days, infiltration returned to the levels detected at 3 days, in both groups (*P* > 0.05). In G3 animals, these levels were significantly increased at 7 days, relative to 3 days (*P* < 0.05). At 14 days, they returned to values similar to those detected at 3 days (*P* > 0.05), but at 21 days values were lower than at both 3 and 14 days (*P* < 0.05).

**Figure 4:**
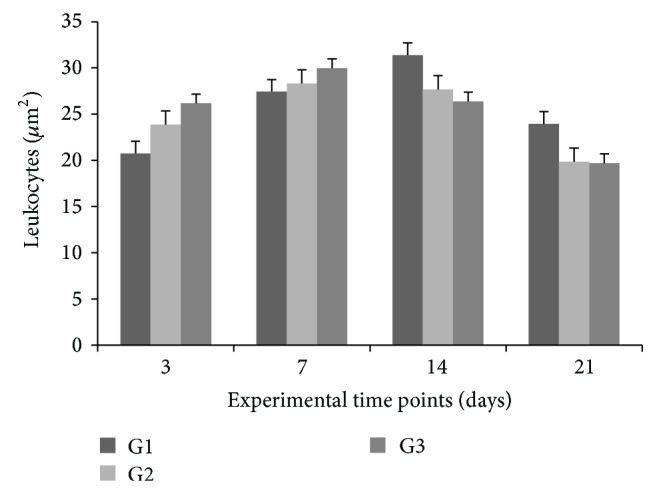
Leukocytic inflammatory infiltrate per optical field at wound site at 3, 7, 14, and 21 days, by group. Values expressed as means ± SEM of group (*n* = 6).

Comparisons across groups showed that at 3 and 7 days the leukocytic inflammatory infiltration levels in G3 were higher than in G1 and G2 (*P* < 0.05), yet with no significant difference (*P* > 0.05) between the latter two groups at these time points. At 14 and 21 days, however, G1 levels were lower than in G2 and G3 (*P* < 0.05), with no significant difference between the latter two groups (*P* > 0.05) (Figure 4).

### 3.5. Total Leukocyte Counts

Group effect, time-point effect, and interaction between these factors (two-way ANOVA for repeated measures, *P* < 0.001) were observed across groups for total leukocyte counts in peripheral blood, relative to baseline values.

Comparisons of time points within each group revealed gradual, significant increases in total leukocyte counts at 7 and 14 days, relative to 3 days (Tukey's post hoc test, *P* < 0.05), but no significant differences between 14 and 21 days (*P* > 0.05). In G2 animals, these levels were significantly increased at 7 days, relative to 3 days (*P* < 0.05). At 14 and 21 days, however, they returned to values similar to those found at 3 days (*P* > 0.05). In G3 animals, total leukocyte counts were significantly increased at 7 days, relative to 3 days (*P* < 0.05). At 14 days, the levels were similar to those found at 3 days (*P* > 0.05), but at 21 days they were lower than at 3 and 7 days (*P* < 0.05).

Comparisons across groups showed that at 3 and 7 days total leukocyte counts were higher in G3 than in G1 and G2 (*P* < 0.05); yet G1 and G2 levels did not differ significantly (*P* > 0.05) at these time points. At 14 days, however, G1 levels were higher than in G2 and G3 (*P* < 0.05), with no significant differences between the latter two groups (*P* > 0.05). At 21 days, these levels were higher in G1 than in G3 (*P* < 0.05); yet the differences between G2 and G3 were not significant (*P* > 0.05) (Table 2).

### 3.6. Mineral Composition of Powdered Shells

**Figure 5:**
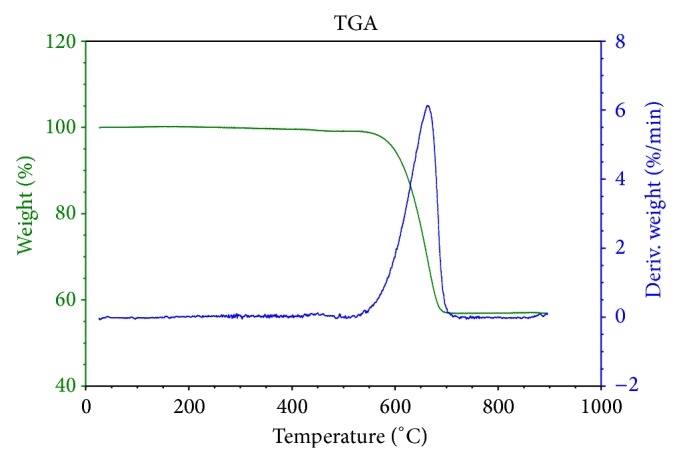
Thermogravimetric analysis of the supernatant in a chloroform solution of* Megalobulimus lopesi* powdered shells.

**Figure 6:**
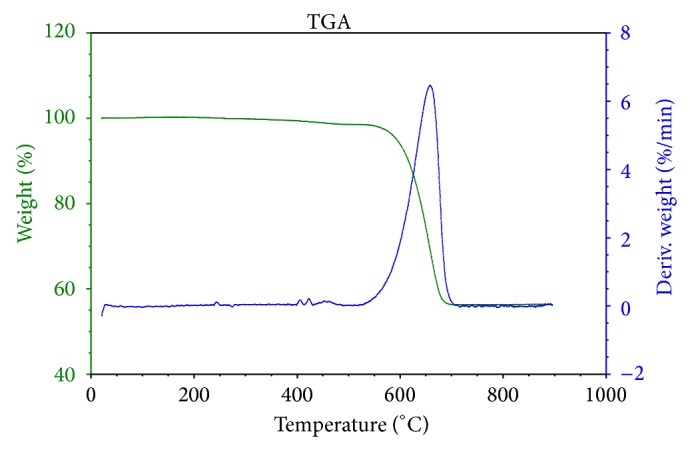
Thermogravimetric analysis of the sediment in a chloroform solution of* Megalobulimus lopesi* powdered shells.

The thermogravimetric curves obtained for the supernatant and the sediment of the chloroform solution of powdered shells are shown in Figures 5 and 6, respectively. For both samples, mass loss profiles typical of calcium carbonate are evident, with a degradation step (550–680°C) that can be attributed to formation of calcium oxide and carbon dioxide (carbonaceous residues).

### 3.7. Organic Composition of Powdered Shells

**Figure 7:**
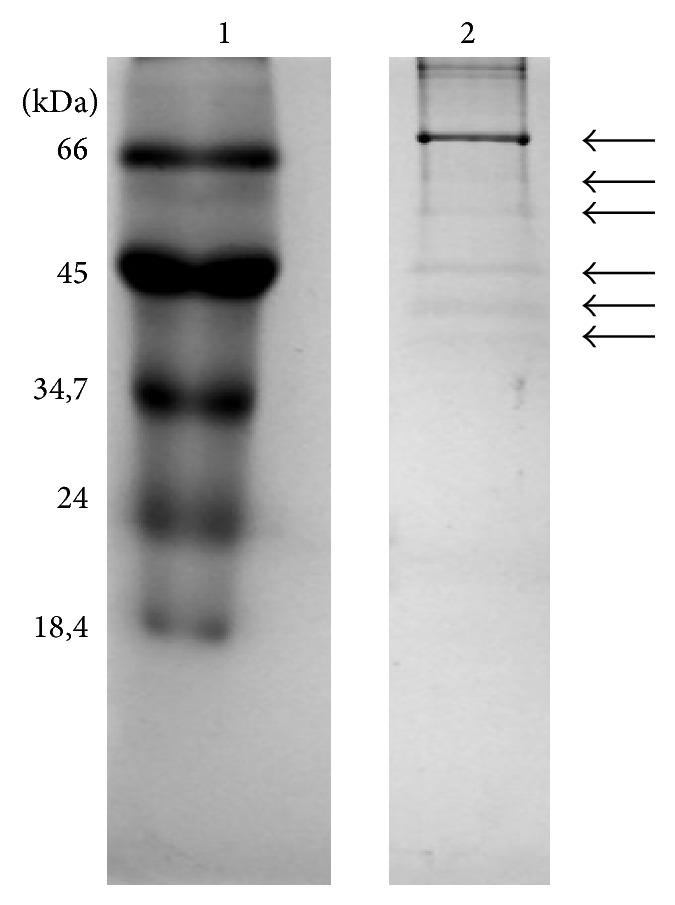
12.5% SDS-PAGE of the sediment of a chloroform solution of* Megalobulimus lopesi* powdered shells. Lane 1 corresponds to molecular weight markers; lane 2 is the sample. The arrows indicate band positions on lane 2.

Protein content was 0.33 *μ*g per milliliter of extract. Six major bands were obtained, of approximately 71.3, 64.7, 59.4, 48.6, 43.4, and 40.7 kDa (Figure 7).

## 4. Discussion

Zootherapy, or the use of animal products for the treatment of human or animal diseases, seems prevalent in certain areas of the world, particularly where traditional medicines are very important, more than allopathic medicine. This is the case for areas such as Brazil, the Middle East, Turkey, China, and Korea. Increasing interest in this field has led to several studies focusing on the historical role of animals in the development of medicine, from either the pharmacological, ethnological, or historical standpoint [36].

Nowadays, there is a great interest for natural products with antibiotic and anticancer activities and a wide range of pharmacological activities from marine animals [37]. Recent studies, aiming at the discovery of novel drugs, have been investigating the therapeutic potential of marine invertebrates (sponges, coelenterates, molluscs, echinoderms, etc.) with very promising results [38]. Moreover, seafood (marine invertebrates and fish) is recognized as a rich source of omega-3 fatty acids and is highly recommended to improve human health and prevent chronic diseases [39].

This was the first study to examine the effect of* M. lopesi* powdered shells using an experimental model of secondary-intention healing. In Asian folk medicine, powdered shells of marine mollusks are often used to treat skin wounds, prevent infection, and ease fever and other symptoms [25]. In several Brazilian regions, mollusk shells are used in the treatment of burns, hemorrhoids, and asthma attacks, as well as in gum irritation and pain during the eruption of primary dentition [27, 40].

In the present study, macroscopic examination of wound areas revealed progressive reduction in animals treated with the powdered shells (G3). Wound area reduction, which consists of wound contraction with approximation of wound edges, is performed by myofibroblasts. Reepithelialization involves margination of keratinocytes by granulation tissue [41]. Full wound contraction was achieved at 21 days in the animals treated with powdered shells (G3), unlike controls (G1) and vehicle controls (G2) at the same time point.

Inflammatory response, a key factor in the process of healing and regeneration, is the early event that shapes the chemotactic gradient, preventing bacterial infection and stimulating debridement of injured tissue by macrophages [12]. In the present investigation, data for leukocytic inflammatory infiltration showed that treatment with powdered shells increased the number of leukocytes in the early stages of wound healing, relative to either of the control groups. Leukocytosis did not persist however, and at 21 days its levels were lower than at 3 days. In both control groups, the increase persisted for up to 14 days; at 21 days, however, the number of leukocytes in the granulation tissue was similar to that found at 3 days.

Excessive inflammatory response can lead to tissue damage, reducing fibroblast proliferation and angiogenesis at the wound site [42]. Nonetheless, modulating the inflammatory process at specific periods of healing progression can be beneficial, particularly in chronic wounds. Topical application of ointment containing powdered shells led to early, but not sustained, leukocytosis.

Inflammation at the wound site elicits concentration of chemical mediators, which tend to enter capillary vessels adjoining the injury and spread throughout the body. In G3 animals, total leukocyte counts in peripheral blood were significantly increased at 3 and 7 days. At 14 and 21 days, however, total leukocyte count was highest in G1 animals, while in G3 animals this variable at 21 days was similar to that found at 3 days.

The mechanisms regulating the early stages of reepithelialization remain unclear, despite evidence of direct involvement of protein kinase C*α* (PKC*α*) and calcium, leading to changes in desmosomal adhesiveness. Desmosomes become calcium-dependent by the action of PKC*α*, enhancing reepithelialization. Compromised reepithelialization has been observed in genetically modified rats lacking PKC*α* genes [43].

In acute wounds, the microenvironment is acidic, with pH ranging from 5 to 6. Acidosis occurs by accumulation of lactic acid, resulting from cellular hypoxia at the wound edges and increased demand for oxygen by local cells [44]. Because CaCO_3_ tends to dissolve under acidic conditions, the ointment containing powdered shells acts as a vehicle for calcium delivery in acidic environments. Calcium administration to the wound bed tends to enhance the healing process [45, 46].

Shells are composed of two phases: one of them is mineral (with CaCO_3_ as the principal constituent); the other, an organic matrix consisting of proteins, glycoproteins, lipids, and polysaccharides, is secreted by the outer mantle, an underlying tissue layer [47]. The proteins in the microstructure of shells have several functions, providing gel or colloidal environments for biomineralization, promoting organized growth and mineral deposition, and performing enzymatic functions, signaling, and patterning for formation of the crystalline mantle [48]. The principal proteins found in* M. lopesi* shells have molecular weights similar to those in mollusks investigated by Berland et al. [49], who reported homogeneity in the molecular weights of shell proteins across mollusk species.

Proteins of 27, 27.84, and 28.96 kDa have been found in shells of* Mytilus edulis* [50], while molecular weights ranging from 24 to 110 kDa and protein concentrations of 1 mg/mL have been reported for* Haliotis rufescens* (Walters et al., 1997), as have proteins of 20 to 200 kDa for* Haliotis asinina* [51]. The present study detected the presence of proteins in the 40 to 70 kDa range. However, the natural variability in molecular weight in proteins from different species is possibly enhanced by differences in protein extraction methods.

Constituents such as enzymes, signaling proteins, glycosaminoglycans, and lipids in the extracellular matrix of mollusk exoskeletons may have biological functions of therapeutic interest for more evolved species, including mammals [52]. In fact, physiological functions of proteins present in invertebrates—more ancient in the evolutionary scale—are identical to those found in humans, despite structural and compositional differences [53].

For instance,* Haliotis laevigata* nacre contains a protein that has shown homology with the N-terminal domain of mammalian insulin-like growth factor binding proteins (IGFBPs). IGF-I and IGF-II are known to participate in the healing process, regulating metabolism and cell growth as well as stimulating collagen synthesis and cell recruitment to injury sites [54]. In the present investigation, the presence of* M. lopesi* shell proteins may have facilitated healing in excision wounds performed in rats.

## 5. Conclusion

This study demonstrated that topical administration of an ointment containing* M. lopesi* powdered shells facilitated secondary-intention healing in an excision wound model in rats. Animals treated with this preparation exhibited decreased wound areas, increased angiogenesis at the wound sites, and increased early inflammatory responses (both locally and systemically), without further tissue damage. These results indicate potential applicability of compositions containing* M. lopesi* powdered shells in secondary-intention healing of cutaneous wounds.

## Figures and Tables

**Table 1:** Progression of wound area, relative to postoperative baseline area, by group.

Groups	3 days	7 days	14 days	21 days
G1	0.659 ± 0.018^bA^	0.472 ± 0.015^bB^	0.392 ± 0.021^aB^	0.207 ± 0.010^aC^
G2	0.783 ± 0.023^aA^	0.634 ± 0.022^aB^	0.378 ± 0.011^bC^	0.129 ± 0.042^bD^
G3	0.533 ± 0.034^cA^	0.430 ± 0.017^bA^	0.204 ± 0.012^cB^	0 ± 0.000^cC^

Values expressed as means ± standard deviations of means. Different lowercase letters in same column indicate significant differences between groups. Different uppercase letters on same row indicate significant differences between time points (Tukey's post hoc test).

**Table 2:** Total leukocyte counts in peripheral blood (cells/mm^3^).

Groups	3 days	7 days	14 days	21 days
G1	11 708 ± 761^bC^	17 508 ± 543^bB^	21 075 ± 871^aA^	17 933 ± 583^aAB^
G2	12 850 ± 848^bB^	19 300 ± 497^bA^	15 666 ± 444^bB^	15 050 ± 678^abB^
G3	18 016 ± 662^aB^	29 000 ± 606^aA^	16 383 ± 583^bBC^	14 858 ± 584^bC^

Values expressed as means ± standard deviations of the means. Different lowercase letters in the same column indicate significant differences between groups. Different uppercase letters on the same row indicate significant differences between time points (Tukey's post hoc test).
